# Moody microbes or fecal phrenology: what do we know about the microbiota-gut-brain axis?

**DOI:** 10.1186/s12916-016-0604-8

**Published:** 2016-04-19

**Authors:** Paul Forsythe, Wolfgang Kunze, John Bienenstock

**Affiliations:** Department of Medicine, McMaster University, Hamilton, Ontario Canada; Department of Psychiatry & Behavioural Neurosciences, McMaster University, Hamilton, Ontario Canada; Department of Pathology & Molecular Medicine, McMaster University, Hamilton, Ontario Canada; McMaster Brain-Body Institute, St. Joseph’s Healthcare, Hamilton, Ontario Canada; Firestone Institute for Respiratory Health, St. Joseph’s Healthcare, Hamilton, Ontario Canada

**Keywords:** Microbiome, Enteric Nervous system, Germ free, Antibiotic, Vagus

## Abstract

**Introduction:**

The microbiota-gut-brain axis is a term that is commonly used and covers a broad set of functions and interactions between the gut microbiome, endocrine, immune and nervous systems and the brain. The field is not much more than a decade old and so large holes exist in our knowledge.

**Discussion:**

At first sight it appears gut microbes are largely responsible for the development, maturation and adult function of the enteric nervous system as well as the blood brain barrier, microglia and many aspects of the central nervous system structure and function. Given the state of the art in this exploding field and the hopes, as well as the skepticism, which have been engendered by its popular appeal, we explore recent examples of evidence in rodents and data derived from studies in humans, which offer insights as to pathways involved. Communication between gut and brain depends on both humoral and nervous connections. Since these are bi-directional and occur through complex communication pathways, it is perhaps not surprising that while striking observations have been reported, they have often either not yet been reproduced or their replication by others has not been successful.

**Conclusions:**

We offer critical and cautionary commentary on the available evidence, and identify gaps in our knowledge that need to be filled so as to achieve translation, where possible, into beneficial application in the clinical setting.

## Introduction

Communication between the gut and brain occurs constantly, largely at a subconscious level, and plays a critical role in maintaining optimal health. Indeed, it is even suggested that defects in gut-brain axis communication are an underlying cause of functional bowel disorders such as irritable bowel syndrome (IBS) [1] and potentially contribute to inflammatory bowel diseases [2]. This gut-brain axis consists of “hard-wired” anatomical connections, involving vagal and spinal nerves, and humoral components provided by the microbiota and their products, gut tissues, endocrine and immune systems.

The gastrointestinal tract, in addition to being the largest endocrine organ, is a nexus of communication between the highest concentration of immune cells in the body, a network of 200-600 million neurons and the trillions of bacteria, fungi and viruses [3] that constitute the human gut microbiota. With this knowledge it seems reasonable to think that intestinal bacteria would influence gut to brain communication and potentially lead to modulation of central nervous system (CNS) function. However, only in the past decade, with advances in sequencing technology, metabolomics and neurophysiology, has the concept of the microbiota-gut -brain axis gained serious attention.

To date there is good evidence that the gut microbiota do play a role in normal CNS development and, in particular, influences systems associated with stress response, anxiety [4–6] and memory [7]. Exposure to certain key commensals can also attenuate the effects of early life stress on CNS development [8, 9]. It is also clear that exposure to specific non-pathogenic bacteria can modulate brain chemistry and behavior in adult animals [10, 11]. Overall, evidence for the existence of a microbiota gut–brain axis is strong. Indeed, growing awareness of the level of integration between host and microbes has led to the suggestion that most living organisms can no longer be considered as individuals; and must be considered holobionts, “whose anatomical, physiological, immunological and developmental functions evolved in shared relationships of different species” [12]. Within the context of the holobiont paradigm, the influence of the gut microbiota over brain development, mood, motivation and behaviour is a natural consequence of the co-evolution of a multi-species organism.

**Fig. 1:**
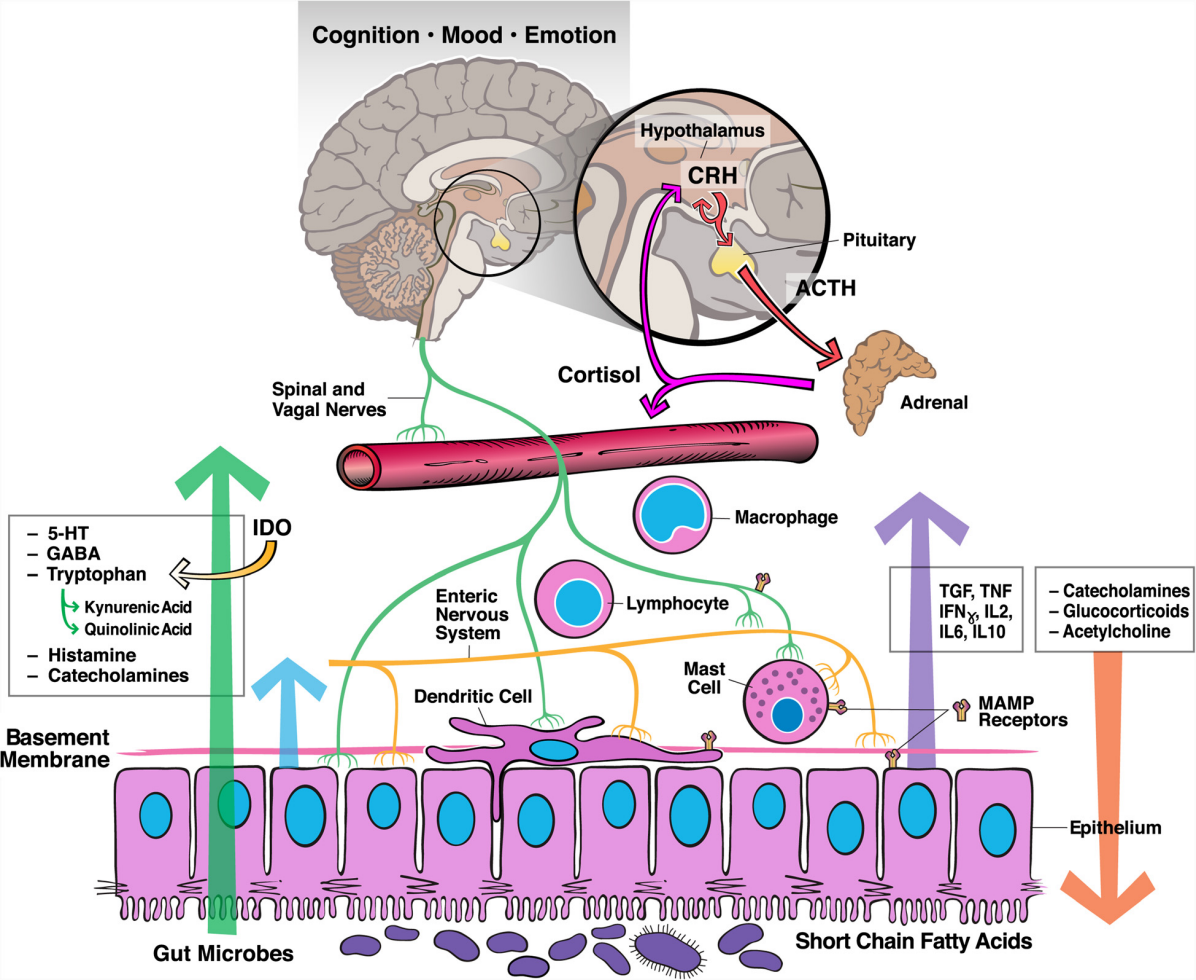
Proposed mechanisms and pathways of the microbiota-gut-brain axis: Gut microbes synthesize a vast array of neuroactive molecules including neurotransmitters such as GABA and through fermentation, short chain fatty acids, which have effects on the nervous system. The intestinal microbiota also has direct and indirect effects of on the intestinal epithelium, local mucosal immune system, enteric nervous system and spinal and vagal nerves. Mediators and signals from these systems, including cytokines and neurotransmitters, modulate central nervous system (CNS) function and neuroendocrine responses such as the hypothalamus pituitary adrenal axis (HPA). In turn signals from the CNS and neuroendocrine system, including cortisol, catecholamines and acetylcholine, can alter gut microbiota composition. While such bi-directional signaling has been identified, definitive evidence for the specific roles of these pathways in communication between gut microbes and the brain is largely lacking

While the potentially paradigm shifting [13] implications of the microbiota-gut-brain axis have garnered much attention in recent years [14–16], we are, nevertheless, at the very early stages of our understanding of this field, and there is limited information, though much speculation, on the complex communication systems involved (Fig. 1).Here we briefly review our current understanding of the mutualistic relationship between gut microbes and the CNS, highlighting recent progress, while identifying gaps in our knowledge and limitations of current methodologies used to explore the microbiota-gut-brain axis.

### Hard-wired connections

**Fig. 2:**
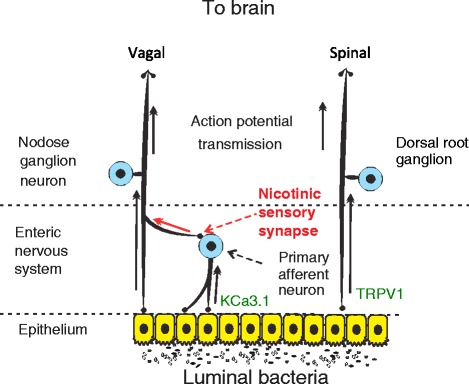
Hardwired connections between gut microbes and the brain: Gut microbes can modulate activity of spinal and vagal sensory neurons. Vagal sensory neuron may assume both primary afferent and interneuron functions via activation of enteric nervous system to vagal fiber nicotinic sensory synapse. Distinct bacterial species have been demonstrated to modulate neural activity through inhibition of the TRPV1 and KCa3.1 ion channels on spinal and intrinsic primary afferents respectively

The major afferent anatomical connections between the gut and the brain include vagal and spinal nerves, their ganglia and the spinal cord. However, within the gut, there are two types of sensory nerves; the extrinsic primary afferent neurons with somata outside the gut, and intrinsic primary afferent neurons (IPANs) with somata within the gut wall. Recent research has identified that certain bacteria and bacterial components in the lumen of the gut can modulate both extrinsic and intrinsic intestinal sensory systems, with consequences for peristalsis, nociception, brain chemistry and mood [10, 11, 17–19] (Fig. 2). Clearly, better knowledge of the neuronal projection pathways by which such signals reach the brain is critical to understanding the microbiota-gut-brain axis. It is also important to emphasize that communication in this axis is bi-directional, and there is strong evidence that, for example, stress has a significant effect on the composition and function of the gut microbiota [20–23].

The gut microbiome is necessary for the normal development of the enteric nervous system [17, 24]. Germ free mice have decreased nerve density, a decreased number of neurons per ganglion, and an increased proportion of myenteric nitrergic neurons in the jejunum and ileum [24]. The changes in neural density are associated with decreased frequency and amplitude of muscle contractions in the jejunum and ileum of germ free mice. A decrease in excitability of myenteric IPANSs has also been demonstrated in germ free animals [17]. Exposing germ-free mice to a normal gut microbiota (conventionalization) normalized both density and activity of enteric neurons [17, 24].

It has been the general understanding that IPANs are primary afferent for gut motility and secretory reflexes while extrinsic sensory neurons are primary afferent for signals to the central nervous system. Until very recently these sensory systems were believed to be separate, with IPANs unable to contribute synaptically to impulses that reach the brain.

However, recent research by Perez-Burgos et al. [25] has identified a nicotinic intramural sensory synapse between the IPANs and the vagus that may have major implications for our understanding of the microbiota-gut-brain axis.

We and others have previously demonstrated that introducing certain bacteria into the gut alters brain chemistry and behavior only if the afferent vagus is intact [10, 11, 18, 26]. Perez-Burgos et al. identified that the majority of the multiunit vagal afferent action potentials evoked by the *L.rhamnosus* strain were dependent on intramural synaptic transmission and were silenced after nicotinic or total synaptic blockade. Furthermore, vagal afferent firing rates correlated monotonically with IPAN excitability, a relationship almost entirely dependent on IPAN/vagal synaptic transmission. The connection between gut microbes, IPANs and the vagus is further supported by the observations that mesenteric afferent signaling following IPAN stimulation is almost completely absent in germ-free mice but restored following conventionalization of the adult animals [27]. While it is not clear how the gut microbiota contribute to normal ENS development and function, alterations in gut microbiota also impact the intestinal and CNS glial cells that provide support and nourishment for neurons as well as regulating synaptic transmission. Kabouridis et al. [28] demonstrated that antibiotic treatment reduced the number of glial cells within the intestinal mucosa, while Erny et al. [29] reported that in germ free mice or conventional mice treated with broad spectrum antibiotics, maturation and function of microglia in the brain was attenuated. It is therefore possible that reduced glial cell numbers and function may underlie at least some of the ENS, and CNS, deficits in GF animals and therefore might underlie the consequences of disturbances in the normal balance of gut microbiota (dysbiosis).

The sheer density of IPANs in the human gut (100 million compared to 50,000 extrinsic sensory vagal and spinal neurons) may make their role as the initial neural sensor of luminal stimuli seem obvious. However, until the study of Perez-Burgos et al. [25] there was no known mechanism whereby sensory signals from these neurons could be communicated to the brain. This work has effectively redrawn the map of hard-wired gut-brain communication suggesting that IPANs relay or gate signals originating from microbes in the lumen to the vagal sensory ganglia. What remains to be identified is how IPAN to vagus nerve gating is regulated, and what determines sensory responses that remain in the gut, such as axon reflexes, versus those that are transmitted to the vagus, and thus communicated to the brain. Furthermore, we do not know how IPANs contribute to the ability of vagal signaling to distinguish between pathogenic and non-pathogenic microbial stimuli [30]. While there is clear evidence that certain ingested pathogenic [31] and non-pathogenic [10, 11, 25] bacteria can modulate vagal activity and subsequently brain chemistry and behavior, to date there is no direct evidence that the vagus nerve is involved in mediating signals from changes in the resident gut microbiota to the brain. Indeed the only study to address this directly, utilizing antibiotics to disrupt the microbiota, suggests that neither the vagus nor the autonomic nervous system is involved [32].

While much of the discussion and research related to neural components of the microbiota-gut-brain axis have focused on the vagus nerve, it is clear the spinal afferents also play a role particularly in relation to microbial modulation of visceral pain perception. Several investigators have shown that ingestion of specific microbes can reduce or inhibit visceral pain induced by gut distension in rodents [33–35]). In the case of one particular organism *Lactobacillus rhamnosus* JB-1, the reduction in visceral pain perception was associated with altered signaling in dorsal root ganglion (DRG) fibres in rats [33].

One of the key receptors responsible for pain perception in the gut is a member of the vanilloid receptor family, the transient receptor potential vanilloid 1 (TRPV1) [36]. The major cellular expression of TRPV1 in the gastrointestinal tract is in spinal and vagal primary afferent neurons [36]. In a recent investigation of the mechanisms underlying the anti-nociceptive activity of *Lactobacillus reuteri* DSM 17938 [37], it was demonstrated that introduction of this organism into the intestinal lumen decreased the firing frequency of nociceptive spinal fibres, but not vagal fibres in the mesenteric nerve bundle. Further investigation revealed that the anti-nociceptive activity of this *L. reuteri* strain likely rests on the ability of the organism to directly or indirectly act as a potent specific blocker of TRPV1 ion channels in extrinsic spinal primary sensory fibres and their corresponding DRG cell bodies. Of note, the antinociceptive activity of the *L.rhamousus* JB-1 strain was independent of TRPV1 antagonism. These findings suggest that the mechanism of action of anti-nociceptive bacteria may differ at the molecular level according to species and strains.

### Microbes and enteroendocrine responses

While neural pathways play an important role in the microbiota-gut-brain axis it is not known how bacteria in the lumen or mucoepithelial layer signal to sensory neurons in the gut. Enteroendocrine, and perhaps other epithelial cells, contain and release a variety of sensory mediators that might activate IPAN, vagal and spinal terminals within the intestine [38]. Putative sensory mediators include serotonin, substance P, somatostatin, CCK, GABA, ATP, and a range of hormones including leptin, orexin, [39, 40]. Two recent studies have identified that components of the normal microbiota regulate host gut serotonergic pathways {Reigstad [41, 42]. Previous studies had established that colonization of germ-free (GF) mice with normal human or mouse derived fecal microbiota significantly accelerates whole-gut transit. These changes in gut motility occur through a mechanism, dependent at least partially, on 5-HT3/4 receptor signaling, suggesting that the gut microbiota may modulate the gut serotonergic pathways of the host [43]. Reigstad et al. [42] utilized GF or humanized (GF colonized with human fecal microbiota) mice to investigate this phenomenon and demonstrated that while human- and mouse-derived complex microbial communities did not alter the sensitivity of the gut to exogenous 5-HT they did increase colonic tryptophan 5-hydroxylase 1 (TPH1) protein and tissue 5-HT concentration. This suggested that increased 5-HT production was responsible for the increase in gut transit time with colonization.

In a separate study, Yano et al. [41] demonstrated that, in particular, spore-forming bacteria (Clostridia species) from the mouse and human microbiota promote 5-HT biosynthesis from colonic enterochromaffin cells (ECs), which supply 5-HT to the mucosa, lumen, and systemically to the bone marrow (circulating platelets). Here, microbiota-dependent effects on gut 5-HT was shown not only to modulate GI motility but also to have implications for host physiology beyond the gut, enhancing platelet activation and aggregation. It is possible these findings are relevant to the microbiota-gut-brain axis, as the modulation of the ENS and gut motility by the EC derived 5-HT may itself influence the composition of gut microbiota, as well as afferent pathways, and consequently brain chemistry and behavior. However the direct consequences of microbiota-induced increases in peripheral 5-HT have yet to be explored in terms of affecting the content and distribution of 5-HT in the brain.

In an attempt to determine the mechanisms underlying the ability of the spore forming bacteria to promote peripheral serotonergic pathways, Yano et al. [41] assessed metabolite level changes in response to conventionalization and identified 47 metabolites that correlated positively with increased 5-HT production. They went on to test the ability of 16 of these metabolites to induce 5-HT in enterochromaffin cell cultures. Among these, α-tocopherol, butyrate, cholate, deoxycholate, p-aminobenzoate, propionate, and tyramine all enhanced 5-HT production [41]. Similarly, Reigstad et al. [42] demonstrated that butyrate and acetate caused concentration-dependent increases in TPH1 expression by an enteroendocrine cell line. Taken together these studies indicate the gut microbiota, and in particular spore forming Clostridia, promote peripheral 5-HT production and that a range of metabolites produced by the microbes can induce 5-HT production *in vitro*. Which, if any, of these metabolites is involved in promoting 5-HT *in vivo* remains to be determined. Furthermore, it is not known if there are any human conditions where a disrupted microbiota leads to modulation of the serotonergic pathways or that modification of conventional microbiota can boost 5-HT production in a physiologically meaningful way.

### Probiotic and prebiotic modulation of the gut-brain axis

To date, the most of the evidence for modulation of the gut-brain axis by microbes comes from assessing changes in brain function and behavior following oral administration of specific bacteria that are often putative probiotics [16]. While an intact vagus seems critical to the ability many of such bacteria to modulate brain chemistry and behavior [10, 44], the underlying mechanisms of action are largely unknown. Host factors and environment, including resident microbiota, likely play an important role in determining the efficacy of “psychoactive” bacteria. Indeed, the ability of *L. helveticus* to attenuate anxiety-like behavior in mice was demonstrated to be genotype and diet-dependent [45].

Specific bacterial strains or even isolated components of microbes are able to acutely modulate both intrinsic and extrinsic gut sensory neuron activity in *ex vivo* preparations suggesting that the modulation of the resident microbiota does not play a major role in mediating their effects [18, 19, 46, 47]. However, a recent *in vivo* study indicates that certain effects of psychoactive bacteria on the brain do require long-term exposure [48]. Mice receiving the psychoactive bacteria *L. rhamnosus* JB-1 for 28 days and were subjected to magnetic resonance spectroscopy weekly and again 4 weeks after cessation of treatment. Glutamate/glutamine levels increased in the brain following 2 weeks of treatment while GABA levels were elevated only after 4 weeks. The fact that no neurotransmitters were elevated before 2 weeks of treatment has parallels with the known delays in the clinical therapeutic effects of antidepressants despite their acute pharmacological actions [49]. Follow up 4 weeks following cessation of treatment revealed that NAA and GABA levels had returned to baseline levels but, significantly, glutamate/glutamine levels remained elevated. This suggests that even though organisms such as *L. rhamnosus* are generally regarded as a transient colonizers and do not persist in the gut, at least some of the effects on brain chemistry may be prolonged.

While experiments utilizing oral treatment of bacteria do provide evidence that microbes can modulate gut-brain communications it is important not to conflate such results with the actions of the resident gut microbiota. It is entirely possible that transient exposure of the entire GI tract to specific organisms triggers mechanisms of immune and neural modulation inaccessible to the permanent bacterial communities/colonizers.

More recently investigators have begun to explore the possibility of modulating gut brain communication by altering resident microbiota through diet, particularly the use of prebiotics that promote the growth of specific potentially beneficial bacteria.

Early support for the suggestion that, in addition to any direct effects of nutritional components, diet-induced changes in bacterial diversity may influence behavior came from a study by Li et al. [50]. Mice fed a diet containing 50 % lean ground beef were found to have a greater diversity of gut bacteria than those receiving standard rodent chow. The increase in bacterial diversity was associated with an increase in working and reference memory and reduced anxiety-like behavior.

More recently a number of studies have investigated the effects of feeding non-digestible oligosaccharides on the CNS. These oligosaccharides pass mainly unabsorbed through the small intestine into the colon and are considered prebiotics as they have been demonstrated to promote the growth of specific gut bacteria including members of the genus Bifidobacterium and Lactobacillus [51, 52]. Mice fed with the human milk oligosaccharides (HMO), 3′Sialyllactose or 6′Sialyllactose, for 2 weeks prior to being exposed to a social disruption stressor were protected against a stressor induced increase in anxiety-like behavior associated changes in gut microbiome profile [53]. Similarly, feeding a mix of non-digestible galacto-oligosaccharides (GOS) was demonstrated to protect against endotoxin induced anxiety-like behavior and to attenuate increases in cortical levels of 5-HT2A receptor and IL-1 β [54].

In unstressed animals, chronic oral administration of the most abundant HMO, 2′-Fucosyllactose, to mice and rats [55] resulted in enhanced hippocampal long-term potentiation (LTP); a process associated with learning memory and fear processing. This effect on LTP was related to better performance of animals in tests of spatial learning, working memory and operant conditioning. In addition, chronic administration of 2′-FL increased the expression of molecules involved in the storage of newly acquired memories, including the postsynaptic density protein 95, phosphorylated calcium/calmodulin-dependent kinase II and brain-derived neurotrophic factor in cortical and subcortical structures.

While such studies may be suggestive of dietary/prebiotic modulation of the microbiota-gut brain axis, it is important to note that the prebiotic oligosaccharides tested all have direct effects on the host independent of the gut microbiota. Sialyllactose is a source of sialic acid which is particularly important for brain development and for cognitive functions. Dietary sialic acid is utilized by brain cells to form gangliosides and sialylated proteins such as neural cell adhesion molecule (NCAM) [56, 57].

Fucosylated HMOs, including 2′-FL, directly diminish colon motor contractions in an *ex vivo* model, suggesting a direct modulation of enteric nerves [58], while GOS have recently been demonstrated to directly modulate the immune system, altering dendritic cell function in vitro [59]. Further studies on the ability of specific oligosaccharides to influence brain chemistry and behavior will be required to discriminate between the microbial contribution of the prebiotic versus direct action on host tissues.

### Antibiotic disruption of the microbiota

The effects of changes in the composition of the gut microbiota on behavior and brain chemistry have also been explored using antibiotic treatments. Bercik et al. [32] demonstrated that in adult BALB/c mice, oral administration of neomycin and bacitracin along with the antifungal agent primaricin lead to a transient change in the composition of the gut microbiota with associated changes in behavior, including increased exploratory drive and decreased apprehension in both the step down and light/dark preference tests. Antibiotic treated animals also had altered BDNF levels in the brain, being decreased in the amygdala while increased in the hippocampus [32]. Furthermore, these investigators showed that fecal transplantation from the NIH Swiss mouse strain (anxiolytic) to the more anxious germfree BALB/c strain within 2 weeks induced in the host the behavioural phenotype of the donor. Similarly, the reverse experiment in which BALB/c provided the donor fecal transplant to the NIH strain also reproduced in the recipients the donor phenotype. The nature of the molecules and/or microbiota responsible for this extraordinary alteration in behavior await exploration.

In a more recent study, Desbonnet el al. [60] tested the behavior of conventionally housed mice treated with a cocktail of antibiotics from weaning (post-natal day 21) onwards. With continuous antibiotic treatment the adult mice exhibited a depleted and restructured gut microbiota together with reduced anxiety-like behavior and cognitive deficits. Changes in behavior in antibiotic treated mice were associated with altered dynamics of the tryptophan metabolic pathway, increased serum tryptophan and decreased kynurenine levels, while analysis of brain chemistry revealed reduced BDNF, oxytocin and vasopressin expression.

As was the case with exposure to specific organisms, it appears that certain antibiotic treatment regimens can also modulate nociception, independently of gut-brain signaling pathways associated with anxiety and cognition. Using a rat model, O’Mahony et al. [61] demonstrated that transient disruption of the gut microbiotia with vancomycin early in life (postnatal day 4 to 13) had no effect on anxiety like behavior or cognitive performance but did lead to long-term increases in visceral hypersensitivity in male but not female animals. Investigators also noted a decrease in the alpha-2 adrenoceptors and TRPV1 in the lumbo-sacral section of the spinal cord of the vancomycin-treated rats in adulthood [61]. This indicates that early exposure to vancomycin leads to permanent alterations in central nervous system pathways. Furthermore, the effect of antibiotic treatment on TRPV1 expression, taken together with evidence that exposure to specific bacteria can modulate activity of this ion channel [37] suggests TRPV1 is a key component of microbial modulation of gut-brain signaling particularly in relation to nociception.

However, overall, rodent studies of the effect of antibiotic administration on the development of visceral hypersensitivity have been contradictory [62, 63]. For example, Rifaximin has been shown to prevent chronic stress-induced visceral hypersensitivity, mucosal inflammation and impaired mucosal barrier function, an effect associated with an increased abundance of Lactobacillus in the ileum of the rats [64]. While the same study showed that neomycin did not prevent visceral hypersensitivity [64]. It is clear that the consequence of antibiotic treatment on physiological responses associated with the gut-brain axis is dependent on the antibiotics given, the animal model used and the timing and duration of treatment in relation to the developmental stage of the animal.

The use of antibiotics may be important tools for investigating the impact of microbiota disruption on mood and behavior. However, caution must be taken when interpreting the results of such studies as the antibiotics themselves may have effects in addition to and unrelated to their antimicrobial actions. Bacitracin, for example, is a protease inhibitor and has been demonstrated to have neurotoxic and/or antinociceptive effects when delivered to the brain [65, 66]. Furthermore, minocycline, a synthetic tetracycline antibiotic, has been shown to exert neuroprotective effects and to delay motor alterations, inflammation and apoptosis in various animal models of neurodegenerative diseases and traumatic brain injury [67, 68]. The neuroprotective effect of minocycline is dependent on the ability of the antibiotic to inhibit activation of microglia [69] and attenuate inducible nitric oxide synthase (iNOS) expression in brain, leading to a decrease in free-radical damage [70]. More recently, minocycline has been demonstrated to protect against malathion induced depression [71] and valproic acid induced autistic behavior [72] in rodents. Furthermore, this antibiotic has been reported to be effective in patients with various psychiatric conditions, but this has yet to be tested in a large randomized clinical trial [73]. While largely untested, it seems highly plausible that certain antibiotics could also have direct effects on neural function when delivered to the “little brain” (enteric nervous system) in the gut. Indeed there is evidence that many antibiotics, including vancomycin, can directly modulate colonic epithelial ion transport ex vivo by depressing neuroepithelial cholinergic neurotransmission [74]. Furthermore, erythromycin has been demonstrated to reduce nerve-mediated intestinal contractions via pre-junctional inhibitory effects on the release of substance P and acetylcholine from nerves in the myenteric plexus of the guinea-pig ileum [75].

In a study by Bercik et al. [32] a causal relationship between antibiotic driven changes in microbiota and behavioral effects was inferred by the demonstration that, in contrast to oral antibiotic treatment, intraperitoneal injection of the antibiotics did not influence behavior. However, their results did not rule out local, direct, effects of antibiotics in the gut or the effects of longer-term administration. Given the extremely high concentrations of antibiotic that have been used to deplete and/or disrupt the microbiota in animal models, a careful investigation of direct effects on the ENS, epithelial cells and immune cells would seem to be prudent. Furthermore, it will be important to conduct studies utilizing clinically relevant doses of antibiotic to identify if “real world” levels of microbiota disruption, particularly during the neonatal developmental period, can have long-term consequences for brain function and behavior.

### Fecal transplants to understand the microbiota-gut-brain axis

Notwithstanding the caveats of antibiotic use described above, antibiotic depletion when combined with fecal transfer a useful tool for exploring the effect of distinct gut microbiota compositions on the gut brain axis. The use of gut microbiota transfer into germ-free animals has been used previously to suggest that distinct behavioral traits of mouse strains may be transferred to germ free mice by the fecal microbiota [32]. However, the experimental use of germ-free mice has limitations. These animals are known to have, inter alia, altered brain chemistry, increased blood brain barrier permeability, an underdeveloped ENS, reduced peripheral 5-HT production, altered gut motility and physiology, and many immune system deficits [5, 6, 17, 41, 42, 76]. How these profound alterations in the physiology of germ-free animals influence the impact of specific transferred organisms is unknown, however fecal bacterial communities transplanted into either normal or germ-free mice do not necessarily replicate the donor community in composition and/or function [77].

Use of antibiotic depletion of the microbiota avoids these limitations of germfree status and the potential direct effects of antibiotics on the host can be mitigated by comparing microbiota with distinct compositions transferred into identically treated control animals. Using such an approach Bruce-Keller et al. [78] demonstrated that mice given the microbiota from donors fed a high fat diet had significant and selective disruptions in exploratory, cognitive, and stereotypical behavior compared with mice receiving control diet microbiota. These behavioral changes occurred in the absence of significant differences in body weight. The high-fat diet microbiota also disrupted markers of intestinal barrier function, increased circulating endotoxin, and increased lymphocyte expression of ICAM-1, TLR-2, and TLR-4. This study does suggest that changes in gut microbiota composition due to dietary changes can influence neurologic function and specifically that the microbiota may contribute to neurologic complications of obesity. However, one potential confounding factor of this study is that animals received by bi-weekly oral gavage with microbiota throughout the experiment. There is the potential that oral exposure to specific organisms within the gavaged microbiota may have effects on the nervous and immune system as they transit through the GI tract, that would not be influenced by changes in composition of resident microorganisms.

### Microbiome profiles in neurodevelopmental and mood disorders

Given the potential for alterations in the gut microbiota to modulate and alter brain function and behavior via neural, immune and endocrine pathways, there has been an increasing interest in determining a microbiome profile, or specific “causal” organisms, associated with behavioural and neuro-developmental conditions such as autism, major depression and even perceived temperament in toddlers [79].

To date the greatest efforts in this area have been directed towards autism spectrum disorders (ASD) with several studies suggesting alterations in gut microbiome composition (reviewed in [80]). However, even here speculation far outstrips good evidence of a causative role for gut microbes (a pubmed search for articles published in a single year (2015) using the terms “autism and microbiome” gave 46 hits, 44 of which were reviews and only 1 an assessment of the microbiome in ASD subjects). While a number of studies have identified differences in the microbiota of ASD versus neurotypical controls, the results are inconsistent (Table 1). Much of the inconsistency may result from differing methodologies used to evaluate the microbiome and the small numbers of subjects in many studies. However, even when similar genome sequencing methods are used, conflicting results have been obtained. Assessment of the three major phyla has revealed decreased [81], increased [82] or unchanged [83, 84] ratios in the relative abundance of Firmicutes and Bacteroidetes together with increased or decreased abundance of Proteobacteria [81–83]. Similarly inconsistent findings have been reported in relation to clostridia and Bifidobacteria [81, 85–87].Furthermore, while many individual studies identify distinct bacteria that appear “abnormal” in ASD, the small number of subjects assessed in many of these studies suggests the potential for type I errors especially in relation to OTUs of relatively low abundance.

**Table 1:**
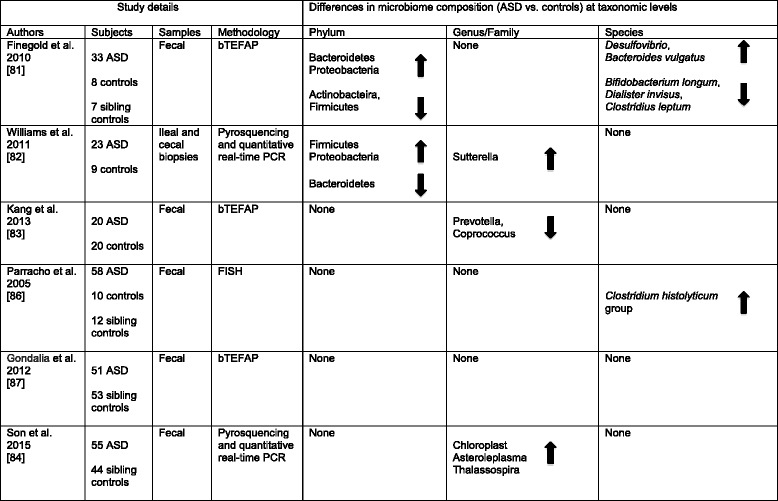
Studies using genome sequencing techniques to determine gut microbiome composition in subjects with Autism Spectrum Disorder (ASD). bTEFAP, Bacterial tag-encoded FLX amplicon pyrosequencing; FISH, Fluorescence in situ hybridization

To date, there have only been only three studies comparing the fecal microbiota of depressed and non-depressed subjects [88–90]. These studies identified an overrepresentation of Bacteroidetes phyla and genus Alistipes but differ in findings related to overall diversity. Furthermore, while Jiang et al. [88] identified a negative correlation between *Faecalibacterium prausnitzii* (FP) and the severity of depression symptoms, Naseribafrouei et al. [89] found no single OTU or clades that correlated with depression. The observations of Szczesniak et al. [90] in addition to their fecal microbiome analysis also showed an increased level of fecal isovaleric acid in depressed patients, pointing out that this was neurotoxic and could cross the blood brain barrier.

Variation in results may occur based on how and when samples are collected and stored, the methods used to extract and sequence DNA and approaches to data analysis. These studies also need to include information on potential confounding factors such as gender, age, use of antibiotics, probiotics or anti-fungals, history of immune disorders including allergy and autoimmune disease, the presence of GI-symptoms and information on diets and use of supplements. Even variables such as stool consistency can have an impact on the microbiome profile measurements [91], and should be taken into account. The selection of the correct control populations is also important. In studies of major depression Jiang et al. [86] used healthy controls while Naseribafrouei et al. [87] recruited outpatients from a neurological units and it has been suggested this may account for the inconsistencies in comparative diversity between those subjects with major depressive disorder and controls in the two studies [88, 89]. Similarly, a recent comparison between ASD patients and neurotypical siblings found no difference in microbiome composition, in contrast to a number of studies examining unrelated controls [84].

Moreover, the majority of studies have been limited to evaluating the microbial community in fecal material, yet studies in animal models and humans indicate that compartment-specific niche differences need to be taken into account in order to understand how the microbiota might impact host health. For example, a study using a restraint stress model in mice demonstrated the differential effect of stress on the composition of the luminal versus mucosa-associated colonic community [92]. The restraint stress induced compartment-specific shifts in the community-wide profile of the colonic microbiota, but only impacted the diversity of the mucosa-associated community. In addition only the mucosa-associated community showed a decrease in the relative abundance of Lactobacillus Sp. Utilizing intestinal biopsies, Williams et al. [82] was able to assess the microbial community of the intestinal mucoepithelial layer in children with autism spectrum disorder and GI disease compared to children with GI disease alone. These authors showed decreases in Bacteroidetes, increases in the ratio of Firmicutes to Bacteroidetes, and increases in Betaproteobacteria; changes that were associated with decreased expression levels of disaccharidases and hexose transporters in intestinal tissue.

While the majority of microbiome studies focus on phyla and class level changes a recent investigation of the gut microbiome of patients with atopic dermatitis opens the possibility that sub-species level changes may have implications for the physiology of the host [93]. Their initial findings were that FP were increased in these patients. This was surprising as reduced abundance of FP had been reported to correlate with disease in patients with Crohn’s disease [94]. Song et al. [93] reported that enrichment of a subspecies of FP that are low producers of butyrate is strongly associated with atopic dermatitis. This intraspecies compositional change in FP reduced the number of high-level producers of butyrate and propionate that have anti-inflammatory effects, and in keeping with this, fecal samples from patients with atopic dermatitis also showed decreased levels of butyrate and propionate. This may be relevant to the microbiota-gut brain axis as short-chain fatty acids (SCFA) such as butyrate are also suggested to play a role in modulating neural function, peripheral 5-HT production and have potent anti-depressant like activity [41, 42, 95, 96].

It is clear that larger studies with more consistent methodology that can be replicated independently, are required if we are to address the issue of whether the gut microbiome of individuals with specific mood or neurodevelopmental disorders are truly different from controls and if so what those differences are, and eventually if these differences are an association or related to causation. The question of causality is complicated by the bi-directional nature of gut-brain signaling. So while behavioral mood or neurodevelopmental disorders may be associated with dysbiosis it is also clear that changes in the CNS impacts the gut microbiota. For example, exposure to psychosocial stress, in addition to altering brain chemistry and inducing anxiety like behavior, also disrupts the gut microbiota. Although, the magnitude and direction of the compositional shift may vary between host genotype and/or models of stress [20–22].

Similarly, exposure to immune challenge or valproic acid, which induce autism-like behavior in mice, also lead to shifts in the microbiome profile [97, 98]. This then begs the question, could host induced changes in the gut microbiota in turn modulate the host? A recent study by De Palma et al. [99] suggests this may be the case. These investigators demonstrated that, in mice, early-life stress alters the colonic microbiota composition, enhances hypothalamic–pituitary–adrenal axis (HPA) and colonic cholinergic neural activity with associated anxiety-like behavior and behavioral despair. Using germ-free mice it was demonstrated that the enhanced HPA and cholinergic activity were independent of the microbiota. However, the microbiota were necessary, but not sufficient, for the induction of behavioral changes, indicating that a combination of host and microbial factors are required.

### Microbial metabolites and the microbiota-gut brain axis

To propose a causal relationship between specific microbes or groups of microbes and mood or neurodevelopmental disorders there should be a viable mechanism through which these microbes might drive changes in brain function and behavior. This might include the production or reduction in the levels of specific metabolites. However, the simple ability of organisms to produce specific metabolites known to be neuromodulators is not enough: they must be shown to have physiologically relevant impact on the levels of these metabolites or to occupy a unique niche within the gut where localized production of these metabolites will influence the host.

There has been much discussion of the roles of short-chain fatty acids (SCFAs) in this regard. Resulting from the fermentation of largely insoluble carbohydrates and fibres by certain components of the gut microbiota, SCFA, mainly acetate, propionate and butyrate are frequently regarded as having a beneficial impact on health. They provide energy for colonocytes, improve ion absorption, have anti-inflammatory properties, and regulate serotonin production and enterochromaffin cell numbers [41, 42, 100]. Furthermore butyrate, an effective histone deacetylase inhibitor, has been reported to have greater antidepressant-like effects than fluoxetine in a mouse model [96]. Conversely, high levels of microbial SCFA production, particularly propionic acid, has been proposed to have detrimental effects on brain development that may lead to autism like behaviors [101]. This has been based on demonstrations that administration of propionic acid by intracerebral injection (or by subcutaneous or oral routes) during key stages of early life development, induces behavioral changes in rodents that correlate with autism [102–104]. The association is further supported by evidence of increased SCFA in stool of autistic children compared with neurotypical controls [105]. However, no assessment has yet been made of whether modulation or disruption of the gut microbiota can result in systemic SCFA levels that are in anyway comparable to the neurotoxic levels achieved following the high (typically 250-500 mg/kg) subcutaneous or oral dose utilized in the rat models [102–104], or that the rat model might reasonably reflect serum levels of SCFA in autistic children.

Nonetheless, a study in an animal model of autism suggests that exposure to specific microbes may attenuate some autism-like behaviors [97]. Injection of pregnant mice with a viral mimic (POLY I:C) resulted in disrupted gut microbiota, increased intestinal permeability and typical stereotypical autistic behaviors in the offspring that lasted into adulthood. Furthermore they identified in a metabolomics approach a molecule, which when administered to naïve mice, largely reproduced the behavioral abnormalities. Oral administration of *B. fragilis* to pregnant mice before and immediately after birth restored microbiota changes, increased intestinal barrier integrity and significantly diminished autistic behaviors. While the administration of *B. fragilis* did not prevent all of the behavioral abnormalities, the findings suggest that viral infection over the course of pregnancy may produce lasting effects, which are potentially reversible by the oral administration of particular bacteria.

The concept of microbial endocrinology [106] has now gained general acceptance. Originally coined in relation to observations that stress, via secretion of catecholamines, could influence the number of potential gut pathogens and influence their proliferation and virulence, the term was extended to include the suggestion that bacterial synthesis of neurotransmitters such as GABA, histamine and 5-HT, plays a role in mediating effects on the gut brain axis with potential impact on mood and behavior [107, 108]. Certainly, evidence that neurotransmitters produced by the host can influence the function of components of the microbiota supports the concept of shared signaling pathways. For example in the QseC sensor kinase of the pathogen Escherichia coli O157:H7 is a receptor for host derived epinephrine/norepinephrine activation which leads to transcription of virulence genes in the bacteria [23, 109]. Conversely there is increasing evidence that signaling molecules of quorum sensing systems, used by bacteria to communicate and coordinate their actions [110], can also bind to mammalian receptors, including taste receptors [111], and directly influence the host [111–113]. A very recent study also indicates that quorum-sensing peptides from Clostridium species can penetrate the blood brain barrier [114]. However, while the concept of microbial endocrinology is attractive and highly plausible, to date there is no evidence that disruption or change of the gut microbiota leads to significant changes in microbe derived neurotransmitters, or that these influence brain function. With the possible exception of histamine mediated effects of *L. reuteri* and *L. rhamnosus* on the immune system [115, 116], there is as yet no evidence that such neurotransmitters influence the microbiota-gut-brain axis.

### Clinical studies

Animal models provide powerful tools for identifying mechanisms of inter-kingdom communication that may influence the microbiota-gut brain axis. For such experiments, rodents, particularly the mouse, are probably the most commonly used models. As with all data obtained from studies in mice, caution must be exercised in extrapolating the potential significance to humans (For a comprehensive review of the strengths and limitations of mouse models in microbiome research see [117]). Specifically, the bi-directional communication between microbiota and host is host specific and thus even humanized mice, may not establish the same relationship with specific organisms as humans. Indeed, it has been concluded that certain taxa resident in the gut microbiota of the human are absent in the humanized mice and mice do not retain a complete profile of transplanted human fecal microbiota [118]. It has also been reported that the immune system of humanized mice does not mature normally [119].

Given the limitations of animal models it is important that we obtain confirmatory evidence of the significance and therapeutic potential of the microbiota-gut-brain axis in human studies. However, to date, there has been very little evidence that disruption of the gut microbiota or exposure to specific non-pathogenic organisms will have the same neurochemical and behavioral effects observed in animal models. In a double-blind, randomized, placebo-controlled study, Messaoudi et al. [120] administered a combination of *L. helveticus* and *Bifidobacterium longum* to healthy women and men for 30 days and then assessed the recipients’ level of anxiety and depression and 24-h urinary-free cortisol levels. Daily administration of the bacteria caused a small but statistically significant improvement in scores related to perceived stress, anxiety and depression compared to placebo. The 24-h urinary cortisol levels were similarly reduced in subjects receiving the bacteria. In another clinical pilot study, 39 patients with a diagnosis of chronic fatigue syndrome were randomly assigned to receive *Lactobacillus casei Shirota* or a placebo daily for 2 months. There was a significant decrease in anxiety, but not depressive symptoms in the treated group [121].

A recent brain imaging study has provided additional impressive supportive evidence for a microbiota-gut-brain axis in humans [122]. This clinical study was performed in 23 healthy women volunteers with no gastrointestinal or psychiatric symptoms. The women were randomly assigned to groups given either a fermented milk product (containing: *Bifidobacterium animalis*, *Streptococcus thermophilus*, *L. bulgaricus*, and *Lactococcus lactis*) or a placebo, which consisted of a nonfermented milk product adjusted for taste and texture, twice daily for 4 weeks. Consumption of the fermented milk product had a robust effect on activity of the brain regions that control central processing of emotion and sensation, as observed with functional magnetic resonance imaging before and after consumption of the fermented milk product.

Another equally interesting study investigated the gut microbiome in a well-characterized group of alcohol-dependent subjects admitted to hospital for withdrawal treatment [123]. A significant subgroup presented with increased intestinal permeability and marked disruption of gut microbiota composition whereas the remainder had gut permeability and microbiota similar to those of healthy controls. Dysbiosis was characterized by reduced levels in the anti-inflammatory bacteria FP and Bifidobacterium and associated with increased scores of depression, anxiety and alcohol craving. After alcohol withdrawal for 3 weeks, all biological markers as well as psychiatric assessments had improved in the patients without marked gut permeability. However, in the patients with increased gut permeability, while this aspect was restored to normal, the dysbiosis and anxiety, depression and craving scores remained. This study strongly suggests that the psychiatric symptoms were primarily associated with the altered gut microbiota.

## Conclusions

There are major gaps in our knowledge regarding how the brain perceives signals from gut bacteria. What is the commensal-induced chemical code triggering ENS and vagal discharge associated with an anxiolytic response? Furthermore, how does the brain process and respond to the multiple signaling pathways relaying information from the microbiota? Consequently, to what extent does disruption of the microbiota or microbe-generated signals contribute to mood, cognition and the development and severity of psychiatric pathology?

Overall, it seems clear that having a gut microbiota is important to the normal development of the central and enteric nervous systems as it also is to essential metabolic activities related to digestion such as appetite, satiety and glucose homeostasis [124]. How qualitative differences in the microbiota might influence neural development and function is less clear. Certainly, the gut microbiota responds to changes in the neurophysiological state of the host, as it responds to changes in diet and circadian rhythm. However we cannot, as yet, claim a profile related to any neurodevelopmental or mood disorder, or ascribe a particular organism or group of organisms to “playing a role” in any such disorders. There is some evidence, in both animal models and humans, that oral exposure to transient organisms can modulate brain chemistry and potentially behavior, but there are no clear indications that altering the resident microbiota would be of therapeutic benefit. There are intense research efforts being made in these areas and it is certain that such studies will help us better understand mental health and the biological underpinnings of mood and neurodevelopmental disorders.

Are we at the whim of our microbes? Or are we expounding “fecal phrenology”? Reality, no doubt, falls somewhere in between, but much more robust, reproducible research, particularly in human subjects, is required before we can confirm the promise of initial experimentation and identify the true implications and therapeutic potential of the microbiota-gut-brain axis.

## References

[1] Wood JD (2002). Neuropathophysiology of irritable bowel syndrome. J Clin Gastroenterol.

[2] Bonaz BL, Bernstein CN (2013). Brain-Gut Interactions in Inflammatory Bowel Diseases. Gastroenterology.

[3] Frank DN, Pace NR (2008). Gastrointestinal microbiology enters the metagenomics era. Curr Opin Gastroenterol.

[4] Sudo N, Chida Y, Aiba Y, Sonoda J, Oyama N, Yu XN (2004). Postnatal microbial colonization programs the hypothalamic-pituitary-adrenal system for stress response in mice. J Physiol.

[5] Heijtz RD, Wang S, Anuar F, Qian Y, Bjorkholm B, Samuelsson A (2011). Normal gut microbiota modulates brain development and behavior. Proc Natl Acad Sci U S A.

[6] Neufeld KM, Kang N, Bienenstock J, Foster JA (2011). Reduced anxiety-like behavior and central neurochemical change in germ-free mice. Neurogastroenterol Motil.

[7] Gareau MG, Wine E, Rodrigues DM, Cho JH, Whary MT, Philpott DJ (2011). Bacterial infection causes stress-induced memory dysfunction in mice. Gut.

[8] Desbonnet L, Garrett L, Clarke G, Kiely B, Cryan JF, Dinan TG (2010). Effects of the probiotic Bifidobacterium infantis in the maternal separation model of depression. Neuroscience.

[9] Gareau MG, Jury J, Macqueen G, Sherman PM, Perdue MH (2007). Probiotic treatment of rat pups normalizes corticosterone release and ameliorates colonic dysfunction induced by maternal separation. Gut.

[10] Bravo JA, Forsythe P, Chew MV, Escaravage E, Savignac HM, Dinan TG (2011). Ingestion of Lactobacillus strain regulates emotional behavior and central GABA receptor expression in a mouse via the vagus nerve. Proc Natl Acad Sci U S A.

[11] Bercik P, Park AJ, Sinclair D, Khoshdel A, Lu J, Huang X (2011). The anxiolytic effect of Bifidobacterium longum NCC3001 involves vagal pathways for gut-brain communication. Neurogastroenterol Motil.

[12] Gilbert SF, Sapp J, Tauber AI (2012). A symbiotic view of life: we have never been individuals. Q Rev Biol.

[13] Mayer EA, Knight R, Mazmanian SK, Cryan JF, Tillisch K (2014). Gut microbes and the brain: paradigm shift in neuroscience. J Neurosci.

[14] Mayer EA, Tillisch K, Gupta A (2015). Gut/brain axis and the microbiota. J Clin Invest.

[15] Borre YE, Moloney RD, Clarke G, Dinan TG, Cryan JF (2014). The impact of microbiota on brain and behavior: mechanisms & therapeutic potential. Adv Exp Med Biol.

[16] Forsythe P, Kunze WA (2013). Voices from within: gut microbes and the CNS. Cell Mol Life Sci.

[17] McVey Neufeld KA, Mao YK, Bienenstock J, Foster JA, Kunze WA (2013). The microbiome is essential for normal gut intrinsic primary afferent neuron excitability in the mouse. Neurogastroenterol Motil.

[18] Perez-Burgos A, Wang B, Mao YK, Mistry B, McVey Neufeld KA, Bienenstock J (2013). Psychoactive bacteria Lactobacillus rhamnosus (JB-1) elicits rapid frequency facilitation in vagal afferents. Am J Physiol Gastrointest Liver Physiol.

[19] Wang B, Mao YK, Diorio C, Wang L, Huizinga JD, Bienenstock J (2010). Lactobacillus reuteri ingestion and IK(Ca) channel blockade have similar effects on rat colon motility and myenteric neurones. Neurogastroenterol Motil.

[20] Bailey MT, Dowd SE, Galley JD, Hufnagle AR, Allen RG, Lyte M (2011). Exposure to a social stressor alters the structure of the intestinal microbiota: implications for stressor-induced immunomodulation. Brain Behav Immun.

[21] Bharwani A, Mian MF, Foster JA, Surette MG, Bienenstock J, Forsythe P (2015). Structural & functional consequences of chronic psychosocial stress on the microbiome & host. Psychoneuroendocrinology.

[22] Bangsgaard Bendtsen KM, Krych L, Sorensen DB, Pang W, Nielsen DS, Josefsen K (2012). Gut microbiota composition is correlated to grid floor induced stress and behavior in the BALB/c mouse. PLoS One.

[23] Freestone PP, Haigh RD, Lyte M (2007). Specificity of catecholamine-induced growth in Escherichia coli O157:H7, Salmonella enterica and Yersinia enterocolitica. FEMS Microbiol Lett.

[24] Collins J, Borojevic R, Verdu EF, Huizinga JD, Ratcliffe EM (2014). Intestinal microbiota influence the early postnatal development of the enteric nervous system. Neurogastroenterol Motil.

[25] Perez-Burgos A, Mao YK, Bienenstock J, Kunze WA (2014). The gut-brain axis rewired: adding a functional vagal nicotinic "sensory synapse". FASEB J.

[26] Mao YK, Kasper DL, Wang B, Forsythe P, Bienenstock J, Kunze WA (2013). Bacteroides fragilis polysaccharide A is necessary and sufficient for acute activation of intestinal sensory neurons. Nat Commun.

[27] McVey Neufeld KA, Perez-Burgos A, Mao YK, Bienenstock J, Kunze WA (2015). The gut microbiome restores intrinsic and extrinsic nerve function in germ-free mice accompanied by changes in calbindin. Neurogastroenterol Motil.

[28] Kabouridis PS, Lasrado R, McCallum S, Chng SH, Snippert HJ, Clevers H (2015). Microbiota controls the homeostasis of glial cells in the gut lamina propria. Neuron.

[29] Erny D, Hrabe de Angelis AL, Jaitin D, Wieghofer P, Staszewski O, David E (2015). Host microbiota constantly control maturation and function of microglia in the CNS. Nat Neurosci.

[30] Forsythe P, Bienenstock J, Kunze WA (2014). Vagal pathways for microbiome-brain-gut axis communication. Adv Exp Med Biol.

[31] Gaykema RP, Goehler LE, Lyte M (2004). Brain response to cecal infection with Campylobacter jejuni: analysis with Fos immunohistochemistry. Brain Behav Immun.

[32] Bercik P, Denou E, Collins J, Jackson W, Lu J, Jury J, et al. The intestinal microbiota affect central levels of brain-derived neurotropic factor and behavior in mice. Gastroenterology. 2011;141:599–609, 609.e1–3. 10.1053/j.gastro.2011.04.052 21683077

[33] Kamiya T, Wang L, Forsythe P, Goettsche G, Mao Y, Wang Y (2006). Inhibitory effects of Lactobacillus reuteri on visceral pain induced by colorectal distension in Sprague-Dawley rats. Gut.

[34] Rousseaux C, Thuru X, Gelot A, Barnich N, Neut C, Dubuquoy L (2007). Lactobacillus acidophilus modulates intestinal pain and induces opioid and cannabinoid receptors. Nat Med.

[35] Duncker SC, Kamiya T, Wang L, Yang P, Bienenstock J (2011). Probiotic Lactobacillus reuteri alleviates the response to gastric distension in rats. J Nutr.

[36] Holzer P (2011). TRP channels in the digestive system. Curr Pharm Biotechnol.

[37] Perez-Burgos A, Wang L, McVey Neufeld KA, Mao YK, Ahmadzai M, Janssen LJ (2015). The TRPV1 channel in rodents is a major target for antinociceptive effect of the probiotic Lactobacillus reuteri DSM 17938. J Physiol.

[38] Blackshaw LA, Brookes SJ, Grundy D, Schemann M (2007). Sensory transmission in the gastrointestinal tract. Neurogastroenterol Motil.

[39] Bertrand PP (2003). ATP and sensory transduction in the enteric nervous system. Neuroscientist.

[40] Ashley Blackshaw L, Young RL (2011). Detection and signaling of glucose in the intestinal mucosa--vagal pathway. Neurogastroenterol Motil.

[41] Yano JM, Yu K, Donaldson GP, Shastri GG, Ann P, Ma L (2015). Indigenous bacteria from the gut microbiota regulate host serotonin biosynthesis. Cell.

[42] Reigstad CS, Salmonson CE, Rainey JF, Szurszewski JH, Linden DR, Sonnenburg JL (2015). Gut microbes promote colonic serotonin production through an effect of short-chain fatty acids on enterochromaffin cells. FASEB J.

[43] Kashyap PC, Marcobal A, Ursell LK, Larauche M, Duboc H, Earle KA (2013). Complex interactions among diet, gastrointestinal transit, and gut microbiota in humanized mice. Gastroenterology.

[44] Bercik P, Verdu EF, Foster JA, Macri J, Potter M, Huang X (2010). Chronic gastrointestinal inflammation induces anxiety-like behavior and alters central nervous system biochemistry in mice. Gastroenterology.

[45] Ohland CL, Kish L, Bell H, Thiesen A, Hotte N, Pankiv E (2013). Effects of Lactobacillus helveticus on murine behavior are dependent on diet and genotype and correlate with alterations in the gut microbiome. Psychoneuroendocrinology.

[46] Al-Nedawi K, Mian MF, Hossain N, Karimi K, Mao YK, Forsythe P (2015). Gut commensal microvesicles reproduce parent bacterial signals to host immune and enteric nervous systems. FASEB J.

[47] Kunze WA, Mao YK, Wang B, Huizinga JD, Ma X, Forsythe P (2009). Lactobacillus reuteri enhances excitability of colonic AH neurons by inhibiting calcium-dependent potassium channel opening. J Cell Mol Med.

[48] Janik R, Thomason LA, Stanisz AM, Forsythe P, Bienenstock J, Stanisz GJ (2016). Magnetic resonance spectroscopy reveals oral Lactobacillus promotion of increases in brain GABA, N-acetyl aspartate and glutamate. Neuroimage.

[49] Frazer A, Benmansour S (2002). Delayed pharmacological effects of antidepressants. Mol Psychiatry.

[50] Li W, Dowd SE, Scurlock B, Acosta-Martinez V, Lyte M (2009). Memory and learning behavior in mice is temporally associated with diet-induced alterations in gut bacteria. Physiol Behav.

[51] Bode L (2012). Human milk oligosaccharides: every baby needs a sugar mama. Glycobiology.

[52] Bruzzese E, Volpicelli M, Squaglia M, Tartaglione A, Guarino A (2006). Impact of prebiotics on human health. Dig Liver Dis.

[53] Tarr AJ, Galley JD, Fisher SE, Chichlowski M, Berg BM, Bailey MT (2015). The prebiotics 3'Sialyllactose and 6'Sialyllactose diminish stressor-induced anxiety-like behavior and colonic microbiota alterations: Evidence for effects on the gut-brain axis. Brain Behav Immun.

[54] Savignac HM, Couch Y, Stratford M, Bannerman DM, Tzortzis G, Anthony DC (2016). Prebiotic administration normalizes lipopolysaccharide (LPS)-induced anxiety and cortical 5-HT2A receptor and IL1-beta levels in male mice. Brain Behav Immun.

[55] Vazquez E, Barranco A, Ramirez M, Gruart A, Delgado-Garcia JM, Martinez-Lara E (2015). Effects of a human milk oligosaccharide, 2'-fucosyllactose, on hippocampal long-term potentiation and learning capabilities in rodents. J Nutr Biochem.

[56] Wang B, Brand-Miller J (2003). The role and potential of sialic acid in human nutrition. Eur J Clin Nutr.

[57] Wang B (2009). Sialic acid is an essential nutrient for brain development and cognition. Annu Rev Nutr.

[58] Bienenstock J, Buck RH, Linke H, Forsythe P, Stanisz AM, Kunze WA (2013). Fucosylated but not sialylated milk oligosaccharides diminish colon motor contractions. PLoS One.

[59] Bermudez-Brito M, Rosch C, Schols HA, Faas MM, de Vos P (2015). Resistant starches differentially stimulate Toll-like receptors and attenuate proinflammatory cytokines in dendritic cells by modulation of intestinal epithelial cells. Mol Nutr Food Res.

[60] Desbonnet L, Clarke G, Traplin A, O'Sullivan O, Crispie F, Moloney RD (2015). Gut microbiota depletion from early adolescence in mice: Implications for brain and behaviour. Brain Behav Immun.

[61] O'Mahony SM, Felice VD, Nally K, Savignac HM, Claesson MJ, Scully P (2014). Disturbance of the gut microbiota in early-life selectively affects visceral pain in adulthood without impacting cognitive or anxiety-related behaviors in male rats. Neuroscience.

[62] Verdu EF, Bercik P, Verma-Gandhu M, Huang XX, Blennerhassett P, Jackson W (2006). Specific probiotic therapy attenuates antibiotic induced visceral hypersensitivity in mice. Gut.

[63] Aguilera M, Cerda-Cuellar M, Martinez V (2015). Antibiotic-induced dysbiosis alters host-bacterial interactions and leads to colonic sensory and motor changes in mice. Gut Microbes.

[64] Xu D, Gao J, Gillilland M, Wu X, Song I, Kao JY (2014). Rifaximin alters intestinal bacteria and prevents stress-induced gut inflammation and visceral hyperalgesia in rats. Gastroenterology.

[65] Herman ZS, Stachura Z, Laskawiec G, Kowalski J, Obuchowicz E (1985). Antinociceptive effects of puromycin and bacitracin. Pol J Pharmacol Pharm.

[66] Yilmaz ER, Gurer B, Kertmen H, Hasturk AE, Evirgen O, Hayirli N (2015). The histopathological and ultrastructural effects of the topical application of bacitracin on the cerebral cortex in rats. Turk Neurosurg.

[67] Tikka TM, Vartiainen NE, Goldsteins G, Oja SS, Andersen PM, Marklund SL (2002). Minocycline prevents neurotoxicity induced by cerebrospinal fluid from patients with motor neurone disease. Brain.

[68] Maegele M (2014). Tetracyclines in traumatic brain injury and sepsis: same, same, but different!*. Crit Care Med.

[69] Tikka T, Fiebich BL, Goldsteins G, Keinanen R, Koistinaho J (2001). Minocycline, a tetracycline derivative, is neuroprotective against excitotoxicity by inhibiting activation and proliferation of microglia. J Neurosci.

[70] Cai ZY, Yan Y, Sun SQ, Zhang J, Huang LG, Yan N (2008). Minocycline attenuates cognitive impairment and restrains oxidative stress in the hippocampus of rats with chronic cerebral hypoperfusion. Neurosci Bull.

[71] Saravi SS, Mousavi SE, Saravi SS, Dehpour AR (2015). Minocycline Attenuates Depressive-Like Behaviour Induced by Rat Model of Testicular Torsion: Involvement of Nitric Oxide Pathway. Basic Clin Pharmacol Toxicol.

[72] Kumar H, Sharma B (1630). Minocycline ameliorates prenatal valproic acid induced autistic behaviour, biochemistry and blood brain barrier impairments in rats. Brain Res.

[73] Carvalho AF, Miskowiak KK, Hyphantis TN, Kohler CA, Alves GS, Bortolato B (2014). Cognitive dysfunction in depression - pathophysiology and novel targets. CNS Neurol Disord Drug Targets.

[74] Goldhill JM, Rose K, Percy WH (1996). Effects of antibiotics on epithelial ion transport in the rabbit distal colon in-vitro. J Pharm Pharmacol.

[75] Minocha A, Galligan JJ (1991). Erythromycin inhibits contractions of nerve-muscle preparations of the guinea pig small intestine. J Pharmacol Exp Ther.

[76] Braniste V, Al-Asmakh M, Kowal C, Anuar F, Abbaspour A, Toth M (2014). The gut microbiota influences blood-brain barrier permeability in mice. Sci Transl Med.

[77] McCafferty J, Muhlbauer M, Gharaibeh RZ, Arthur JC, Perez-Chanona E, Sha W (2013). Stochastic changes over time and not founder effects drive cage effects in microbial community assembly in a mouse model. ISME J.

[78] Bruce-Keller AJ, Salbaum JM, Luo M, Blanchard E, Taylor CM, Welsh DA (2015). Obese-type gut microbiota induce neurobehavioral changes in the absence of obesity. Biol Psychiatry.

[79] Christian LM, Galley JD, Hade EM, Schoppe-Sullivan S, Kamp Dush C, Bailey MT (2015). Gut microbiome composition is associated with temperament during early childhood. Brain Behav Immun.

[80] Mayer EA, Padua D, Tillisch K (2014). Altered brain-gut axis in autism: comorbidity or causative mechanisms?. Bioessays.

[81] Finegold SM, Dowd SE, Gontcharova V, Liu C, Henley KE, Wolcott RD (2010). Pyrosequencing study of fecal microflora of autistic and control children. Anaerobe.

[82] Williams BL, Hornig M, Buie T, Bauman ML, Cho Paik M, Wick I (2011). Impaired carbohydrate digestion and transport and mucosal dysbiosis in the intestines of children with autism and gastrointestinal disturbances. PLoS One.

[83] Kang DW, Park JG, Ilhan ZE, Wallstrom G, Labaer J, Adams JB (2013). Reduced incidence of Prevotella and other fermenters in intestinal microflora of autistic children. PLoS One.

[84] Son JS, Zheng LJ, Rowehl LM, Tian X, Zhang Y, Zhu W (2015). Comparison of Fecal Microbiota in Children with Autism Spectrum Disorders and Neurotypical Siblings in the Simons Simplex Collection. PLoS One.

[85] Song Y, Liu C, Finegold SM (2004). Real-time PCR quantitation of clostridia in feces of autistic children. Appl Environ Microbiol.

[86] Parracho HM, Bingham MO, Gibson GR, McCartney AL (2005). Differences between the gut microflora of children with autistic spectrum disorders and that of healthy children. J Med Microbiol.

[87] Gondalia SV, Palombo EA, Knowles SR, Cox SB, Meyer D, Austin DW (2012). Molecular characterisation of gastrointestinal microbiota of children with autism (with and without gastrointestinal dysfunction) and their neurotypical siblings. Autism Res.

[88] Jiang H, Ling Z, Zhang Y, Mao H, Ma Z, Yin Y (2015). Altered fecal microbiota composition in patients with major depressive disorder. Brain Behav Immun.

[89] Naseribafrouei A, Hestad K, Avershina E, Sekelja M, Linlokken A, Wilson R (2014). Correlation between the human fecal microbiota and depression. Neurogastroenterol Motil.

[90] Szczesniak O, Hestad K, Hanssen JF, Rudi K. Isovaleric acid in stool correlates with human depression. Nutr Neurosci. 2015. [Epub ahead of print] 10.1179/1476830515Y.0000000007 25710209

[91] Vandeputte D, Falony G, Vieira-Silva S, Tito RY, Joossens M, Raes J (2016). Stool consistency is strongly associated with gut microbiota richness and composition, enterotypes and bacterial growth rates. Gut.

[92] Galley JD, Yu Z, Kumar P, Dowd SE, Lyte M, Bailey MT (2014). The structures of the colonic mucosa-associated and luminal microbial communities are distinct and differentially affected by a prolonged murine stressor. Gut Microbes.

[93] Song H, Yoo Y, Hwang J, Na YC, Kim HS. Faecalibacterium prausnitzii subspecies-level dysbiosis in the human gut microbiome underlying atopic dermatitis. J Allergy Clin Immunol. 2015. Epub ahead of print] 10.1016/j.jaci.2015.08.021 26431583

[94] Sokol H, Pigneur B, Watterlot L, Lakhdari O, Bermudez-Humaran LG, Gratadoux JJ (2008). Faecalibacterium prausnitzii is an anti-inflammatory commensal bacterium identified by gut microbiota analysis of Crohn disease patients. Proc Natl Acad Sci U S A.

[95] Kimura I, Inoue D, Maeda T, Hara T, Ichimura A, Miyauchi S (2011). Short-chain fatty acids and ketones directly regulate sympathetic nervous system via G protein-coupled receptor 41 (GPR41). Proc Natl Acad Sci U S A.

[96] Schroeder FA, Lin CL, Crusio WE, Akbarian S (2007). Antidepressant-like effects of the histone deacetylase inhibitor, sodium butyrate, in the mouse. Biol Psychiatry.

[97] Hsiao EY, McBride SW, Hsien S, Sharon G, Hyde ER, McCue T (2013). Microbiota modulate behavioral and physiological abnormalities associated with neurodevelopmental disorders. Cell.

[98] de Theije CG, Wopereis H, Ramadan M, van Eijndthoven T, Lambert J, Knol J (2014). Altered gut microbiota and activity in a murine model of autism spectrum disorders. Brain Behav Immun.

[99] De Palma G, Blennerhassett P, Lu J, Deng Y, Park AJ, Green W (2015). Microbiota and host determinants of behavioural phenotype in maternally separated mice. Nat Commun.

[100] Scheppach W, Christl SU, Bartram HP, Richter F, Kasper H (1997). Effects of short-chain fatty acids on the inflamed colonic mucosa. Scand J Gastroenterol Suppl.

[101] MacFabe DF (2015). Enteric short-chain fatty acids: microbial messengers of metabolism, mitochondria, and mind: implications in autism spectrum disorders. Microb Ecol Health Dis.

[102] MacFabe DF, Cain DP, Rodriguez-Capote K, Franklin AE, Hoffman JE, Boon F (2007). Neurobiological effects of intraventricular propionic acid in rats: possible role of short chain fatty acids on the pathogenesis and characteristics of autism spectrum disorders. Behav Brain Res.

[103] Ossenkopp KP, Foley KA, Gibson J, Fudge MA, Kavaliers M, Cain DP (2012). Systemic treatment with the enteric bacterial fermentation product, propionic acid, produces both conditioned taste avoidance and conditioned place avoidance in rats. Behav Brain Res.

[104] El-Ansary AK, Ben Bacha A, Kotb M (2012). Etiology of autistic features: the persisting neurotoxic effects of propionic acid. J Neuroinflammation.

[105] Wang L, Christophersen CT, Sorich MJ, Gerber JP, Angley MT, Conlon MA (2012). Elevated fecal short chain fatty acid and ammonia concentrations in children with autism spectrum disorder. Dig Dis Sci.

[106] Freestone PP, Sandrini SM, Haigh RD, Lyte M (2008). Microbial endocrinology: how stress influences susceptibility to infection. Trends Microbiol.

[107] Lyte M (2011). Probiotics function mechanistically as delivery vehicles for neuroactive compounds: Microbial endocrinology in the design and use of probiotics. Bioessays.

[108] Dinan TG, Stanton C, Cryan JF (2013). Psychobiotics: a novel class of psychotropic. Biol Psychiatry.

[109] Clarke MB, Hughes DT, Zhu C, Boedeker EC, Sperandio V (2006). The QseC sensor kinase: a bacterial adrenergic receptor. Proc Natl Acad Sci U S A.

[110] Hughes DT, Sperandio V (2008). Inter-kingdom signalling: communication between bacteria and their hosts. Nat Rev Microbiol.

[111] Saunders CJ, Christensen M, Finger TE, Tizzano M (2014). Cholinergic neurotransmission links solitary chemosensory cells to nasal inflammation. Proc Natl Acad Sci U S A.

[112] Boontham P, Robins A, Chandran P, Pritchard D, Camara M, Williams P (2008). Significant immunomodulatory effects of Pseudomonas aeruginosa quorum-sensing signal molecules: possible link in human sepsis. Clin Sci (Lond).

[113] Telford G, Wheeler D, Williams P, Tomkins PT, Appleby P, Sewell H (1998). The Pseudomonas aeruginosa quorum-sensing signal molecule N-(3-oxododecanoyl)-L-homoserine lactone has immunomodulatory activity. Infect Immun.

[114] Wynendaele E, Verbeke F, Stalmans S, Gevaert B, Janssens Y, Van De Wiele C (2015). Quorum Sensing Peptides Selectively Penetrate the Blood-Brain Barrier. PLoS One.

[115] Thomas CM, Hong T, van Pijkeren JP, Hemarajata P, Trinh DV, Hu W (2012). Histamine derived from probiotic Lactobacillus reuteri suppresses TNF via modulation of PKA and ERK signaling. PLoS One.

[116] Frei R, Ferstl R, Konieczna P, Ziegler M, Simon T, Rugeles TM (2013). Histamine receptor 2 modifies dendritic cell responses to microbial ligands. J Allergy Clin Immunol.

[117] Nguyen TL, Vieira-Silva S, Liston A, Raes J (2015). How informative is the mouse for human gut microbiota research?. Dis Model Mech.

[118] Turnbaugh PJ, Ridaura VK, Faith JJ, Rey FE, Knight R, Gordon JI (2009). The effect of diet on the human gut microbiome: a metagenomic analysis in humanized gnotobiotic mice. Sci Transl Med.

[119] Chung H, Pamp SJ, Hill JA, Surana NK, Edelman SM, Troy EB (2012). Gut immune maturation depends on colonization with a host-specific microbiota. Cell.

[120] Messaoudi M, Lalonde R, Violle N, Javelot H, Desor D, Nejdi A (2011). Assessment of psychotropic-like properties of a probiotic formulation (Lactobacillus helveticus R0052 and Bifidobacterium longum R0175) in rats and human subjects. Br J Nutr.

[121] Rao AV, Bested AC, Beaulne TM, Katzman MA, Iorio C, Berardi JM (2009). A randomized, double-blind, placebo-controlled pilot study of a probiotic in emotional symptoms of chronic fatigue syndrome. Gut Pathog.

[122] Tillisch K, Labus J, Kilpatrick L, Jiang Z, Stains J, Ebrat B (2013). Consumption of fermented milk product with probiotic modulates brain activity. Gastroenterology.

[123] Leclercq S, Matamoros S, Cani PD, Neyrinck AM, Jamar F, Starkel P (2014). Intestinal permeability, gut-bacterial dysbiosis, and behavioral markers of alcohol-dependence severity. Proc Natl Acad Sci U S A.

[124] De Vadder F, Kovatcheva-Datchary P, Goncalves D, Vinera J, Zitoun C, Duchampt A (2014). Microbiota-generated metabolites promote metabolic benefits via gut-brain neural circuits. Cell.

